# Origin and evolution of signaling pathways responsible for ascorbic acid synthesis and catabolism during plant terrestrialization

**DOI:** 10.1093/hr/uhaf184

**Published:** 2025-07-23

**Authors:** Li-Yao Su, Zheng-Tai Liu, Pei-Yan Chen, Xi-Liang Wang, Hui Liu, Jin-Song Xiong, Ai-Sheng Xiong

**Affiliations:** State Key Laboratory of Crop Genetics and Germplasm Enhancement and Utilization, Ministry of Agriculture and Rural Affairs Key Laboratory of Biology and Germplasm Enhancement of Horticultural Crops in East China, College of Horticulture, Nanjing Agricultural University, Nanjing, 210095 Jiangsu, China; State Key Laboratory of Crop Genetics and Germplasm Enhancement and Utilization, Ministry of Agriculture and Rural Affairs Key Laboratory of Biology and Germplasm Enhancement of Horticultural Crops in East China, College of Horticulture, Nanjing Agricultural University, Nanjing, 210095 Jiangsu, China; State Key Laboratory of Crop Genetics and Germplasm Enhancement and Utilization, Ministry of Agriculture and Rural Affairs Key Laboratory of Biology and Germplasm Enhancement of Horticultural Crops in East China, College of Horticulture, Nanjing Agricultural University, Nanjing, 210095 Jiangsu, China; State Key Laboratory of Crop Genetics and Germplasm Enhancement and Utilization, Ministry of Agriculture and Rural Affairs Key Laboratory of Biology and Germplasm Enhancement of Horticultural Crops in East China, College of Horticulture, Nanjing Agricultural University, Nanjing, 210095 Jiangsu, China; State Key Laboratory of Crop Genetics and Germplasm Enhancement and Utilization, Ministry of Agriculture and Rural Affairs Key Laboratory of Biology and Germplasm Enhancement of Horticultural Crops in East China, College of Horticulture, Nanjing Agricultural University, Nanjing, 210095 Jiangsu, China; State Key Laboratory of Crop Genetics and Germplasm Enhancement and Utilization, Ministry of Agriculture and Rural Affairs Key Laboratory of Biology and Germplasm Enhancement of Horticultural Crops in East China, College of Horticulture, Nanjing Agricultural University, Nanjing, 210095 Jiangsu, China; State Key Laboratory of Crop Genetics and Germplasm Enhancement and Utilization, Ministry of Agriculture and Rural Affairs Key Laboratory of Biology and Germplasm Enhancement of Horticultural Crops in East China, College of Horticulture, Nanjing Agricultural University, Nanjing, 210095 Jiangsu, China

## Abstract

This study comprehensively reveals the origin and evolution mechanisms of ascorbic acid (AsA) synthesis and breakdown pathways during plants’ transition from water to land. By analyzing genomic data from 21 key plant species and transcriptomic data from the One Thousand Plants transcription project, we found that the L-galactose pathway emerged in green algae, with variations in the HIT domain of the rate-limiting enzyme GGP driving adaptive divergence between lower and higher plants. The galacturonic acid pathway integrated with the L-galactose pathway through the emergence of GalUR in bryophytes. The *myo*-inositol pathway became complete in bryophytes, and its refinement likely promoted dehydration adaptation via oxidative protection. The AsA recycling pathway (APX/MDHAR/DHAR) originated in red algae, while the appearance of AO enzymes is significantly related to rising oxygen levels during land colonization. Statistical analysis of 218 plant species shows that AsA content increases significantly with evolution, in line with heightened light and oxygen stress. This study explains the dynamic evolution of the AsA metabolic network during plant terrestrialization, highlighting how key gene families (e.g. GGP, GalUR, GLOase) undergo functional and structural domain divergence to boost antioxidant capacity and thus facilitate adaptation to terrestrial life. These findings offer a theoretical basis for improving crop stress resistance.

## Introduction

Ascorbic acid (AsA), a core antioxidant in plants, plays extensive roles in growth, development, environmental adaptation, and diverse physiological processes. Its primary function lies in antioxidant defense, where AsA serves as a critical scavenger of reactive oxygen species (ROS), maintaining redox homeostasis to mitigate oxidative damage under drought, high temperature, and high-light stress [1]. Under high-light conditions, AsA synergizes with catalase (CAT) and glutathione (GSH) to alleviate oxidative injury [2]. AsA is indispensable in photoprotection, where it scavenges excess ROS generated by Photosystem II (PSII), preserves the structural integrity of photosynthetic membranes, and modulates the synthesis and degradation of photosynthetic pigments, thereby sustaining photosynthetic efficiency [3]. AsA regulates cell wall biosynthesis through dynamic changes in its redox status, promoting cell wall loosening and expansion to facilitate cellular growth [4]. During fruit ripening, oxidation-dependent shifts in AsA levels are directly linked to cell wall softening, while the activity of ascorbate oxidase (AO) further modulates cell wall integrity, influencing fruit texture [5]. As an enzymatic cofactor, AsA drives multiple biochemical reactions. For example, it is an essential cofactor for 2-oxoglutarate-dependent dioxygenases, participating in the biosynthesis of plant hormones (e.g. ethylene and gibberellins) and secondary metabolites [6], while also promoting iron reduction and uptake [7]. In growth regulation, AsA influences cell cycle progression by modulating redox signaling, thereby governing cell division and expansion [8]. Studies demonstrate that dynamic fluctuations in AsA levels during fruit development correlate with fruit size, texture, and ripening timing [8]. AsA contributes to plant stress adaptation by interacting with hormone signaling pathways [9]. In summary, the multifunctional properties of AsA establish it as a pivotal molecule in plant physiology and stress adaptation.

The transition of plants from aquatic to terrestrial environments represents a pivotal evolutionary milestone, marked by multifaceted environmental challenges including fluctuating light intensity, drought, and oxidative stress. To adapt to these pressures, plants evolved diverse physiological mechanisms, among which the central role of AsA has become increasingly prominent. In terms of light adaptation, terrestrial plants face photo-oxidative stress caused by fluctuating light intensities, where excessive high light induces the overaccumulation of ROS, leading to cellular structural damage [10]. As a potent antioxidant, AsA can directly scavenges ROS [2], and it can also enhance the efficiency of light energy utilization by participating in the electron transfer process during photosynthesis [3], thereby balancing photoprotection and energy capture. Under drought stress, AsA enhances plant adaptability through dual mechanisms: on one hand, it regulates cellular osmotic pressure to maintain water homeostasis [11], and on the other hand, it improves drought resistance by modulating hormone signaling pathways, such as abscisic acid-mediated responses [12]. Regarding oxidative stress, AsA constitutes a central component of the antioxidant defense system. Its role extends beyond direct ROS neutralization to include activation of antioxidant enzyme systems, such as superoxide dismutase (SOD) [1], thereby protecting proteins, lipids, and DNA from oxidative damage [2].

From a metabolic regulation perspective, the biosynthetic pathway of AsA has undergone significant evolutionary refinement during plant terrestrialization. Key enzymes in its synthesis, such as L-galactose dehydrogenase, exhibit high conservation from green algae to higher plants [13], indicating selective advantages of this pathway for terrestrial adaptation. Environmental factors, including ultraviolet radiation and drought, dynamically regulate the expression of AsA biosynthesis genes [10], and such metabolic plasticity enables plants to rapidly respond to environmental fluctuations. AsA serves as a pleiotropic molecule in plant terrestrial adaptation through its antioxidant properties, osmoregulatory functions, and integration into metabolic networks. The regulatory differences of AsA in different plant lineages and the molecular basis of its evolutionary adaptability remain unclear.

AsA, a vital antioxidant and cofactor in plants, displays marked diversity in its synthesis and metabolic pathways among plant lineages. The most well-characterized biosynthetic route is the L-galactose pathway, which converts glucose-6-phosphate into AsA via a series of enzymatic reactions [14]. GDP-L-galactose phosphorylase (GGP), the rate-limiting enzyme in this pathway, is dynamically regulated by environmental and developmental cues [15, 16]. Enzymes such as L-galactose-1-phosphate phosphatase (GPP) and L-galactose dehydrogenase catalyze critical steps in this pathway [14]. Beyond the L-galactose pathway, the *myo*-inositol pathway may contribute to AsA synthesis through intermediate conversion, though its mechanistic details and quantitative significance remain unverified [17]. The L-gulose pathway potentially acts as an auxiliary route in certain plant species, but its universality and functional relevance are yet to be fully resolved [18, 19]. Metabolically, AsA exists in reduced and oxidized forms, with their dynamic equilibrium being essential for cellular redox homeostasis. This balance is maintained by monodehydroascorbate reductase (MDHAR) and dehydroascorbate reductase (DHAR), where DHAR utilizes GSH as a cofactor to reduce DHA via the glutathione-ascorbate cycle [18, 19].

Comparative studies reveal significant differences in AsA content among plant lineages, with green algae, bryophytes, and ferns generally exhibiting lower AsA levels than seed plants [11]. This divergence may correlate with evolutionary adaptation strategies: seed plants have enhanced AsA biosynthetic capacity to cope with more complex oxidative stress environments, whereas lower plants, predominantly inhabiting humid habitats, face relatively reduced antioxidant demands [11]. AsA is extensively involved in plant growth, development, and stress responses. Environmental factors such as light intensity positively regulate AsA biosynthesis, while drought and high temperature accelerate its oxidative consumption [15, 16]. At the functional level, AsA not only acts as an antioxidant to scavenge ROS but also participates in the biosynthesis and signaling of phytohormones, including ethylene and abscisic acid [12]. In summary, investigations into the synthesis, metabolism, and multifunctional roles of AsA provide critical insights into plant adaptation mechanisms and the regulatory networks of antioxidant systems.

The transition of plants from aquatic to terrestrial environments represents a pivotal milestone in the history of life, marked by adaptive responses to multifaceted environmental challenges, including fluctuating light intensity, drought, and oxidative stress. In these complex habitats, AsA emerged as a central antioxidant, playing an indispensable role in plant resilience to diverse abiotic stresses. As plants evolved to colonize terrestrial ecosystems, the biosynthetic and catabolic pathways of AsA underwent significant evolutionary refinement and optimization, establishing it as a hallmark of adaptive evolution. However, the origins and evolutionary mechanisms underlying these metabolic signaling pathways during plant terrestrialization remain poorly understood. Advancing research in this field will not only deepen our understanding of AsA’s biological functions but also elucidate how plants dynamically remodel metabolic networks to adapt to shifting environmental conditions. This research therefore aims to explore the evolutionary origins and diversification of AsA synthesis and degradation pathways during plant terrestrialization. By identifying and analyzing key evolutionary divergences across plant lineages, we seek to provide novel insights into the molecular basis of plant adaptive evolution. This work may inform future genetic engineering strategies to enhance crop stress tolerance by leveraging conserved or lineage-specific features of AsA metabolism.

## Results

### Identification of core elements in the plant AsA biosynthesis pathway

In this study, we focus on elucidating the signaling pathways governing AsA biosynthesis during plant terrestrialization. Specifically, we prioritize four core pathways: the L-galactose pathway, galacturonate pathway, *myo*-inositol pathway, and recycling pathway. The L-gulose pathway was excluded from analysis due to limited prior evidence or presumed functional redundancy in terrestrial adaptation.

To systematically identify AsA biosynthesis homologs, we selected 14 regulatory genes implicated in AsA synthesis across plants. Genomic annotations from 21 representative species (spanning red algae to angiosperms; see Table S1) were analyzed to trace evolutionary trajectories of these genes. Furthermore, we leveraged transcriptomic data from the One Thousand Plants (1KP) initiative, encompassing ~1000 species, to perform large-scale screening of AsA biosynthesis homologs. This dual approach combining targeted genomic analysis with broad transcriptomic surveys enables robust identification of gene origins and homology across evolutionary timescales.

### The origin of the L-galactose pathway in plants

**Figure 1:**
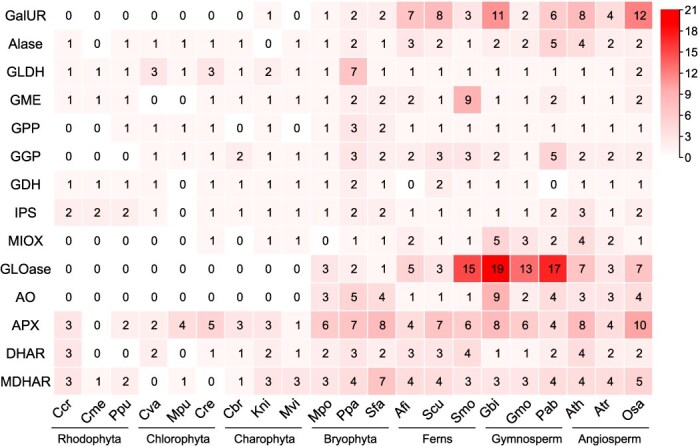
The distribution of ascorbate synthesis and metabolism gene homologs among 21 representative species from seven lineages, with color intensity indicating gene copy number. Ath: *Arabidopsis thaliana*; Atr: *Amborella trichopoda*; Osa: *Oryza sativa*; Gbi: *Ginkgo biloba*; Gmo: *Gnetum montanum*; Pab: *Picea abies*; Afi: *Azolla filiculoides*; Scu: *Salvinia cucullata*; Smo: *Selaginella moellendorffii*; Mpo: *Marchantia polymorpha*; Ppa: *Physcomitrium patens*; Sfa: *Sphagnum fallax*; Cba: *Chara braunii*; Kni: *Klebsormidium nitens*; Mvi: *Mesostigma viride*; Cva: *Chlorella variabilis*; Mpu: *Micromonas pusilla*; Cre: *Chlamydomonas reinhardtii*; Ccr: *Chondrus crispus*; Cme: *Cyanidioschyzon merolae*; Ppu: *Porphyridium purpureum*.

**Figure 2:**
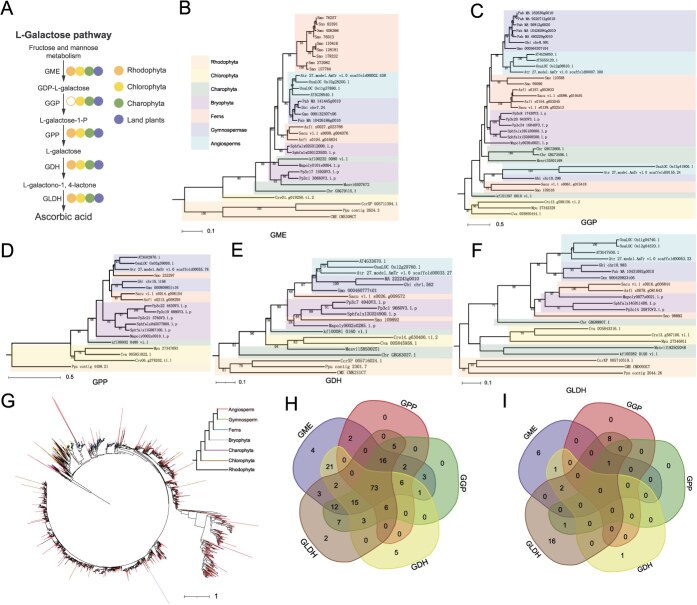
The L-galactose pathway originated in Chlorophyta. (A) Schematic representation of the L-galactose pathway for AsA biosynthesis from fructose and mannose derivatives. Circles from left to right represent Rhodophyta, Chlorophyta, Charophyta, and land plants. Solid circles indicate the presence of a gene in the corresponding lineage, while hollow circles denote absence. (B–F) Phylogenetic trees of gene family members for GME, GGP, GPP, GDH, and GLDH across 21 core plant species. Background colors distinguish major plant lineages. (G) Phylogenetic tree of GGP gene family members derived from the 1KP transcriptome database. The coloration of clades can be used to distinguish different plant groups. (H, I) Clustering analysis of GME, GGP, GPP, GDH, and GLDH homologs in chlorophytes and glaucophytes based on the 1KP dataset.

The L-galactose pathway, as the primary route for AsA biosynthesis in most plants, provides critical insights into the evolutionary origins and functional diversification of AsA in plant adaptation. In this study, we investigated five key enzymes within this pathway: GDP-mannose 3,5-epimerase (GME), GGP, GPP, L-galactose dehydrogenase (GDH), and L-galactono-1,4-lactone dehydrogenase (GLDH). Genomic analysis of 21 species revealed that GME, GPP, GDH, and GLDH were ubiquitously distributed across all seven major lineages examined (Figs 1 and 2B, D–F), with *Porphyridium purpureum* representing the earliest diverging organism harboring all four genes. Transcriptomic screening of the 1KP dataset further confirmed the widespread presence of these genes across lineages (Figs S1–S4). For GGP, 35 homologs were identified in the 21 genomes (Figs 1 and 2C). The earliest GGP homologs emerged in Chlorophyta, while *Chlamydomonas reinhardtii* was the first organism possessing all five pathway genes. Phylogenetic analysis revealed that GME, GPP, GDH, and GLDH clustered according to plant evolutionary relationships. In contrast, six GGP homologs from divergent species formed a basal clade (Fig. 2C), suggesting functional divergence during plant evolution. Among the 35 GGP homologs, 10 lacked nuclear localization sequences, and three lacked histidine triad (HIT) domains. Strikingly, homologs from *Oryza sativa*, *Amborella trichopoda*, and *Physcomitrium patens* exhibited concurrent loss of both domains, contradicting previous reports (Fig. S5). Transcriptomic analysis of 1KP data detected abundant GGP homologs in all lineages except Rhodophyta, with homologs also identified in glaucophytes (Fig. 2G). To trace the origin of a complete L-galactose pathway, we assessed gene presence in glaucophytes and chlorophytes. While 73 chlorophyte species retained all pathway genes, only *P. atomus* (*Picochlorum atomus*) among glaucophytes harbored GGP, GLDH, GME, and GPP (Fig. 2H and I). Multiple sequence alignment revealed divergence in the HIT domain of glaucophyte GGP homologs: whereas land plants conserve an HxHxQ motif (x = hydrophobic residue), glaucophyte homologs frequently exhibited nonhydrophobic substitutions at these positions (Fig. S6), implying functional differentiation. In summary, during algal evolution, glaucophytes, rhodophytes, and chlorophytes diverged as sister lineages. Rhodophytes subsequently lost GGP, while a complete L-galactose pathway originated in the chlorophyte lineage (Fig. 2A).

### The origin of the galacturonate pathway in plants

**Figure 3:**
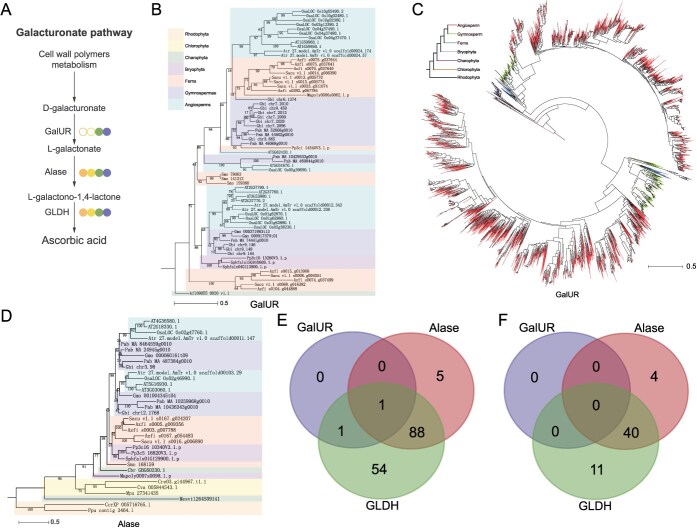
The galacturonate pathway originated in Chlorophyta. (A) Schematic representation of the galacturonate pathway for AsA biosynthesis from cell wall degradation products. Symbols follow Fig. 2A. (B, D) Phylogenetic trees of GalUR and Alase gene family members across 21 core plant species. Background colors denote major plant lineages. (C) Phylogenetic tree of GalUR homologs derived from the 1KP transcriptome database. The coloration of clades can be used to distinguish different plant groups. (E, F) Clustering analysis of GalUR, Alase, and GLDH gene homologs in chlorophytes and glaucophytes based on the 1KP dataset.

The galacturonate pathway, a secondary route for AsA biosynthesis in plants, plays a pivotal role in linking cell wall metabolism with antioxidant production. In this pathway, cell wall-derived metabolites are converted into D-galacturonate via undefined reactions, which is subsequently catalyzed by galacturonate reductase (GalUR) and aldonolactonase (Alase) to converge with the L-galactose pathway. Among the 21 selected species, GalUR was first identified in the charophyte *Klebsormidium nitens*, while *Alase* homologs were distributed across all seven major lineages (Fig. 1). Phylogenetic analysis of 46 GalUR homologs revealed that this gene originated in charophytes and subsequently diversified during plant evolution (Fig. 3B). Transcriptomic screening of the 1KP dataset further identified GalUR homologs in Charophyta, with phylogenetic divergence coinciding with algal terrestrialization (Fig. 3C). For Alase, phylogenetic reconstruction using genomic and 1KP transcriptomic data demonstrated its early origin, with homologs prevalent in Rhodophyta and members of the Chromista (Fig. 3D and Fig. S7). Additionally, we found that the Alase gene members underwent a duplication event in seed plants. To investigate whether this event led to functional divergence, we performed a multiple sequence alignment of the conserved domain (L-ascorbate oxidase: TIGR03388) among Alase protein members. The results revealed significant divergence in this domain between red algae and other plant lineages. During evolution, the L-ascorbate oxidase domain underwent continuous changes, but its sequence remained largely conserved between green algae and land plants (Fig. S8). Chlorophytes harbored 2 GalUR, 94 Alase, and 144 GLDH homologs, including co-occurring GalUR and Alase in *I. paradoxum* (*Interfilum paradoxum*) (Fig. 3E). Notably, Rhodophyta and SAR lineages exhibited abundant Alase and GLDH homologs (Fig. 3F). These findings collectively suggest that Alase and GLDH originated in the common ancestor of Archaeplastida, while the complete galacturonate pathway emerged concurrently with GalUR acquisition in chlorophytes (Fig. 3A).

### The origin of the *myo*-inositol pathway in plants

**Figure 4:**
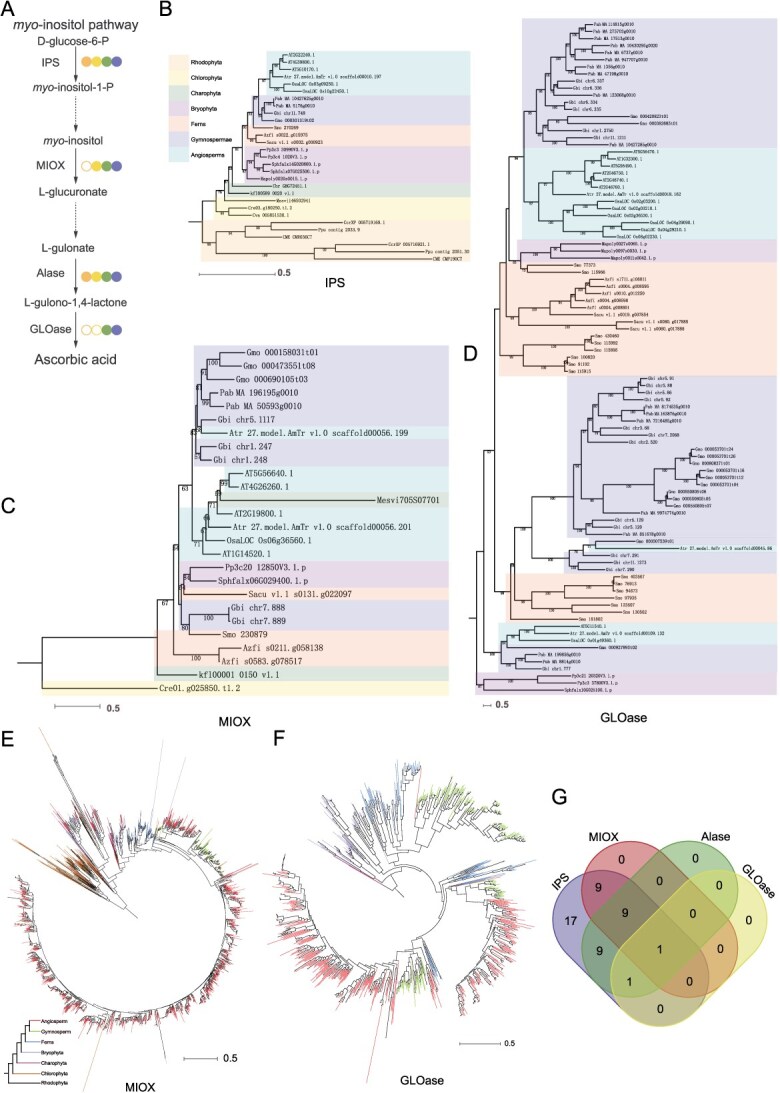
The *myo*-inositol pathway originated in Charophytes. (A) Schematic representation of the *myo*-inositol pathway for AsA biosynthesis from inositol. Symbols follow Fig. 2A. (B–D) Phylogenetic trees of IPS, MIOX, and GLOase protein members across 21 core plant species. Background colors distinguish major plant lineages. (E, F) Phylogenetic trees of MIOX and GLOase homologs derived from the 1KP transcriptome database. The coloration of clades can be used to distinguish different plant groups. (G) Clustering analysis of IPS, MIOX, Alase, and GLOase homologs in chlorophytes and glaucophytes based on the 1KP dataset.

The *myo*-inositol pathway synthesizes AsA from inositol through sequential catalysis by inositol phosphate synthase (IPS), *myo*-inositol oxygenase (MIOX), *Alase*, and gulonolactone oxidase (GLOase). Among the 21 species analyzed, IPS homologs were universally present across all lineages (Fig. 1). Phylogenetic reconstruction of IPS homologs revealed evolutionary trajectories congruent with plant phylogeny (Fig. 4B). Transcriptomic screening of the 1KP dataset further confirmed abundant IPS homologs in Rhodophyta (Fig. S9). MIOX homologs first emerged in chlorophytes (Fig. 4C and E), with no evidence of functional divergence during evolution. In contrast, GLOase homologs identified in the 21 species exhibited diversification into three distinct clades (Fig. 4D). Further extraction and multiple sequence alignment of the functional domains (FAD-binding domain (PF01565) and D-arabinono-1,4-lactone oxidase domain (PF04030)) of GLOase members were performed. The results revealed that the FAD-binding domain is highly conserved in plants (Fig. S10A), whereas the D-arabinono-1,4-lactone oxidase domain in bryophytes is significantly shorter than that in seed plants. Additionally, the sequence of this domain in *P. patens*) exhibits extremely low similarity to that in other plants (Fig. S10B). Analysis of 1KP data revealed that GLOase originated in charophytes, subsequently splitting into three lineages during fern evolution, with one lineage lost in angiosperms (Fig. 4F). Notably, the charophyte *Spirotaenia* sp*.* was identified as the earliest lineage possessing a complete *myo*-inositol pathway, evidenced by co-occurring IPS, MIOX, Alase, and GLOase homologs (Fig. 4G). Collectively, these results demonstrate that the complete *myo*-inositol pathway arose in charophytes, and its functional integrity depends critically on GLOase.

### The origin of the AsA recycling pathway in plants

**Figure 5:**
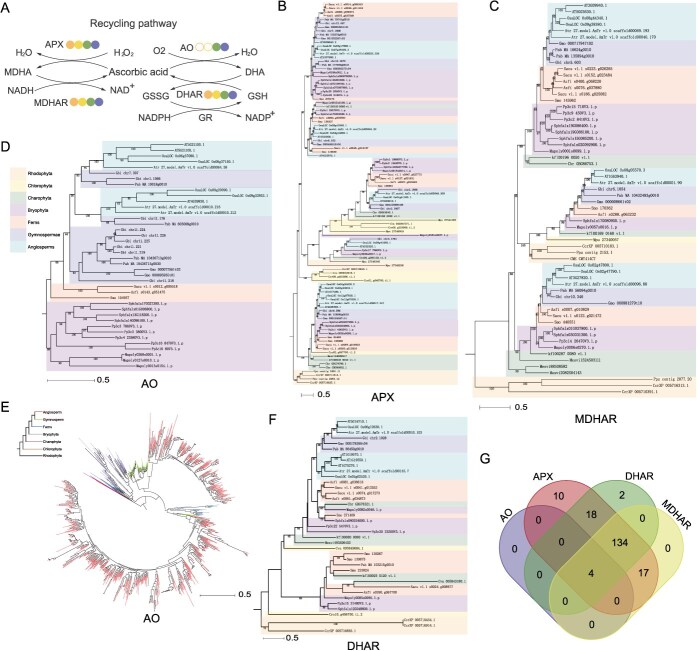
The ascorbate recycling pathway originated prior to red algae. (A) Schematic of the ascorbate recycling pathway (legend follows Fig. 2A). (B–D, F) Phylogenetic trees of APX, MDHAR, AO, and DHAR gene family members across 21 core plant species. Background colors denote distinct plant taxonomic groups. (E) Phylogenetic tree of AO protein members reconstructed using the 1KP transcriptome database. The coloration of clades can be used to distinguish different plant groups. (G) Clustering analysis of APX, MDHAR, AO, and DHAR orthologs in green algae and glaucophytes based on 1KP transcriptome data.

The ascorbate recycling pathway plays a crucial role in plants, where AO and ascorbate peroxidase (APX) utilize AsA as both an antioxidant and electron donor to continuously scavenge ROS and regenerate NAD^+^ without depleting AsA reserves (Fig. 5A). Among 21 selected species, we identified 101 APX genes distributed across all major plant lineages (Figs 1 and 5B). Functional divergence of APX genes initially emerged in red algae (Fig. 5B), with 1KP transcriptome data further confirming this differentiation. The four ancestral clades of extant land plant APX genes originated in green algae, while Charophyceae developed the complete five ancestral clades (Fig. S11). Additionally, 62 MDHAR genes were identified in these genomes (Fig. 1). Phylogenetic analysis revealed that MDHAR underwent functional divergence in red algae (Fig. 5C), though transcriptomic evidence indicates this differentiation occurred in green algae, with three ancestral clades emerging in Charophyceae (Fig. S12).

AO originated in Charophyceae and underwent functional diversification beginning in ferns (Fig. 5D and E). DHAR, a key enzyme linking ascorbate and glutathione cycles (Fig. 5A), first appeared in red algae but achieved functional diversification in angiosperms (Fig. 5F and Fig. S13). While MDHAR can be generated through non-enzymatic reactions, the DHA-AsA recycling pathway strictly requires APX, MDHAR, and DHAR. The emergence of AO likely represents an evolutionary adaptation to enhance environmental adaptability by converting oxygen to water through AsA oxidation and promoting DHA-to-AsA conversion via glutathione cycling. Notably, numerous orthologs of APX, MDHAR, and DHAR were identified in red algae (Fig. S14A) and Chromista (Fig. S14B), whereas complete ortholog sets of AO, APX, MDHAR, and DHAR were only detected in four Charophyceae species (Fig. 5G). These findings suggest that the core APX-MDHAR-DHAR-mediated ascorbate recycling pathway originated earlier in eukaryotic red algae. The later emergence of AO may represent an adaptive innovation to cope with elevated atmospheric oxygen levels during plant terrestrialization, facilitating enhanced DHA-to-AsA conversion through intensified glutathione cycling.

### Synthesis and metabolism of AsA in relation to light and oxygen stress in the plant growth environment

**Figure 6:**
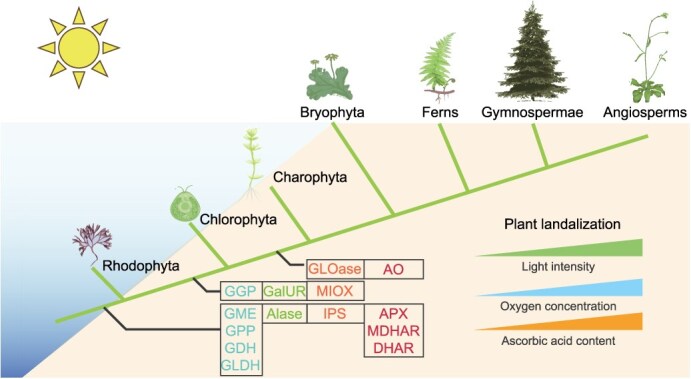
Schematic diagram depicting the evolution of core AsA biosynthetic machinery during plant terrestrialization, showing ancestral deep-sea algae lacking complete pathways, refinement during the transition to shallow water under increasing light/oxygen, and augmented accumulation adapting plants to terrestrial stressors.

In this study, we focused on investigating the evolutionary trajectory of AsA synthesis and metabolism pathways during plant terrestrialization and their interplay with environmental factors. Although the L-gulose pathway may play a significant role in certain plant lineages, it was excluded from our analysis due to its incomplete representation across most species. Through systematic collection and statistical analysis of AsA content in 218 plant species (Table S2), we observed a significant evolutionary trend of increasing AsA levels, which correlates closely with elevated light intensity and atmospheric oxygen concentrations during plant terrestrialization. As a pivotal antioxidant, AsA plays a crucial role in photosynthesis and oxygen stress responses. We thus propose that rising light and oxygen levels acted as key environmental drivers shaping the evolutionary adaptation of AsA synthesis and metabolism pathways. By systematically identifying and screening orthologs of core regulatory genes, we reconstructed a putative evolutionary model (Fig. 6) to elucidate the mechanistic links between AsA pathway evolution and plant terrestrialization.

## Discussion

The AsA synthesis and metabolic pathways in plants represent pivotal evolutionary innovations enabling environmental adaptation. In this study, we systematically identified orthologs of core regulatory genes associated with AsA biosynthetic pathways, including the L-galactose pathway, galacturonate pathway, and *myo*-inositol pathway. The evolutionary origins and diversification patterns of these pathways illuminate the intricate adaptive mechanisms underlying plant terrestrialization.

The L-galactose pathway serves as the primary route for AsA biosynthesis in plants, with its evolutionary origin traceable to green algae [18, 20]. In green algae, key enzymes of the L-galactose pathway including GME, GPP, GDH, and GLDH were already present, while the GGP gene first emerged in Chlorophyta [10]. The evolutionary trajectory of these genes aligns with plant phylogeny, indicating that the L-galactose pathway was progressively refined during plant terrestrialization. The HIT motif, as a crucial functional motif in GGP members, features a conserved sequence (HxHxQx) in which the histidine residues serve as catalytic active sites [21]. This motif mediates the phosphorolytic cleavage of GDP-L-galactose to produce L-galactose-1-phosphate, a key step in AsA biosynthesis [22]. Mutations of the histidine residues within the HIT motif disrupt both substrate-binding and enzymatic activity [23]. Functional divergence of GGP, particularly variations in its HIT domain, likely reflects adaptive divergence between early-diverging lineages (e.g. glaucophytes) and vascular plants [24, 25].

The galacturonate pathway, mediated by the enzymes GalUR and Alase, converts cell wall metabolites into D-galacturonate, which is subsequently integrated into the L-galactose pathway [19]. Research indicates that the charophyte *K. nitens* is the earliest species to possess GalUR, while Alase is widely distributed across multiple plant lineages, suggesting its origin in an ancient unicellular ancestor [10]. Notably, this pathway establishes a modular connection between cell wall metabolites (D-galacturonate) and the L-galactose pathway, potentially providing metabolic plasticity that enables plants to cope with terrestrial environmental stresses. The integration of these functions may have laid the foundation for the evolution of multicellular plant metabolic pathways.

The *myo*-inositol pathway utilizes *myo*-inositol as a precursor and generates AsA through the catalytic actions of IPS, MIOX, Alase, and GLOase. Research suggests that charophytes, a close relative of green plants, may represent the earliest photosynthetic eukaryotes to possess a complete *myo*-inositol pathway [10]. Notably, Alase homologs are already present in rhodophytes (an independent lineage) and chromalveolates, indicating that this gene likely originated during the early stages of eukaryotic photosynthesis [18]. Given the charophytes’ role as the sister group to land plant ancestors, the refinement of the *myo*-inositol pathway may have contributed to the adaptation of green plants to terrestrial environments characterized by dehydration and high radiation levels, potentially through ascorbate-mediated oxidative protection mechanisms. Meanwhile, we observed the evolution of the D-arabinono-1, 4-lactone oxidase domain in the GLOase protein. The D-arabinono-1,4-lactone oxidase domain is the key catalytic domain that exhibits absolute substrate specificity [26]. The GLOase protein first emerged in bryophytes; however, the D-arabinono-1, 4-lactone oxidase domain in bryophytes shows significant differences compared to that in land plants. Therefore, we hypothesize that it plays a crucial role in maintaining normal physiological functions and antioxidant defense mechanisms in bryophytes [27–29]. Additionally, the affinity or catalytic efficiency of bryophyte GLOase for substrates such as D-arabinono-1,4-lactone may differ from that of land plants, leading to variations in the efficiency and mode of action of AsA synthesis under different substrate conditions.

Alase is a shared protein between the galacturonate pathway and the *myo*-inositol pathway. Alase belongs strictly to a large superfamily of strictosidine synthase-like proteins. It is widely distributed in both plants and animals and exhibits broad substrate specificity [30–32]. We observed the evolution of the Alase functional domain (PTHR23075) demarcated by bryophytes. This domain in bryophytes and algae differs from that in ferns and seed plants. The terrestrial environment is characterized by significantly enhanced ultraviolet radiation and oxidative stress, which likely increased the demand for AsA as a critical antioxidant [33–35]. Studies in *P. patens* suggest that Alase likely functions primarily by facilitating the degradation of dehydroascorbate rather than promoting ascorbate biosynthesis [36]. The functional role of Alase in the ascorbate biosynthesis pathway may have been enhanced in ferns and seed plants, driving the directional evolution of the Alase domain. The conservation of the Alase domain likely signifies that this enzyme underwent critical functional innovation or optimization during plant terrestrialization to meet the demands of terrestrial environments.

The AsA recycling pathway plays a crucial role in plants, with core enzymes including APX, MDHAR, DHAR, and AO. These enzymes are essential for regenerating AsA, ensuring its sustained capacity to scavenge ROS [37]. This study reveals that the origins of APX, MDHAR, and DHAR can be traced back to rhodophytes, while AO originated in charophytes [10]. The evolutionary patterns of these enzymes suggest that the AsA recycling pathway was gradually refined during plant terrestrialization to adapt to increasing oxygen concentrations in terrestrial environments. Notably, the emergence of AO may have been driven by the need to enhance the conversion of dehydroascorbate to AsA, thereby strengthening the glutathione cycle in response to rising oxygen levels during terrestrialization [10].

During the terrestrialization of plants, the biosynthesis and metabolic pathways of AsA underwent significant evolution to adapt to increased light intensity and oxygen concentrations in terrestrial environments. This study systematically collected and analyzed AsA levels across 218 plant species, revealing a significant upward trend in AsA content with plant evolution [10]. This trend is closely associated with the increased light intensity and external oxygen concentrations during terrestrialization. Elevated light and oxygen levels enhance ROS production in plant cells, while AsA, as a key antioxidant, effectively scavenges ROS, thereby protecting plant cells from oxidative damage [1, 11]. Previous studies have demonstrated that conspecific plants cultivated at high-altitude environments exhibit significantly elevated AsA content compared to their low-altitude counterparts [38, 39]. This altitudinal disparity in antioxidant accumulation may be attributed to intensified solar radiation exposure at higher elevations, with photoinhibition-induced oxidative stress potentially serving as a critical environmental inducer for enhanced AsA biosynthesis and subsequent adaptive responses. Hormones serve as essential bioactive substances for plant growth and development. Previous studies have revealed the origin and evolution of multiple plant hormone signaling pathways, demonstrating their close association with plant terrestrialization [40–43]. Extensive research has shown that plant hormones play a significant role in regulating ascorbic acid biosynthesis [44–47]. Therefore, the emergence of endogenous plant hormones may have been crucial for the evolution of ascorbic acid synthesis.

In plant terrestrialization, AsA biosynthesis and metabolism pathways significantly evolved to adapt to terrestrial environments, enhancing antioxidant capacity and promoting survival and reproduction. Future research could explore the specific functions of these pathways in different plant lineages, relevant hormone roles, and environmental adaptation mechanisms.

## Materials and methods

### Identification of members of AsA synthesis and metabolism genes in plants

We performed BLASTP analysis using *Arabidopsis thaliana* genes involved in ascorbate biosynthesis and metabolism as queries against annotated genes from 21 representative plants across seven lineages (Table S1) and the 1KP transcriptome data, with an E-value cut-off of 1e−5. The genomic data were sourced from public databases, including Phytozome and PlantGIR [48], among others. The resulting sequences were subsequently annotated using InterProScan software [49], and gene family members were identified according to the criteria established in Table S3. Phylogenetic trees for each gene family were constructed using FastTree [50] and visualized through the iTOL web platform [51]. Members containing anomalous clades underwent additional validation. Ultimately, we comprehensively identified all putative genes participating in ascorbate biosynthesis and metabolism pathways from both the 21 representative plant species and the 1KP transcriptome dataset.

### Phylogenetic tree construction of AsA synthesis and metabolism gene family members

To investigate the evolutionary origins of ascorbate biosynthesis and metabolism genes, we first reconstructed phylogenetic trees for these gene families. Multiple sequence alignments were generated for each gene family using MAFFT with parameters -thread 20, -maxiterate 1000, and -localpair [52]. The resulting alignments were subsequently trimmed using Phyutility with the -clean 0.5 threshold to remove poorly aligned regions [53]. Optimal amino acid substitution models were determined through ProtTest under the criteria -all-distributions -F -AIC -BIC -tc 0.5 -threads 20 [54]. Maximum-likelihood phylogenetic trees were then constructed using IQ-TREE2 with model specifications detailed in Table S4, employing parameters -B 1000 (1000 ultrafast bootstrap replicates) and -t 20 (20 parallel threads) [55]. Final tree visualization and annotation were performed using the iTOL web platform [51].

## Compliance with ethics requirement

This article does not contain any studies with human or animal subjects.

## Supplementary Material

Web_Material_uhaf184

## Date availability

Data are available in figures of the article and its supplementary materials.
