# Supplementary material for: Origin and evolution of signaling pathways responsible for ascorbic acid synthesis and catabolism during plant terrestrialization
Source: Hortic Res. 2025 Jul 23;12(10):uhaf184. doi: 10.1093/hr/uhaf184 (PMC12537018; doi:10.1093/hr/uhaf184)
Supplement: Web_Material_uhaf184 [file web_material_uhaf184.zip › Supplementary figures.docx]

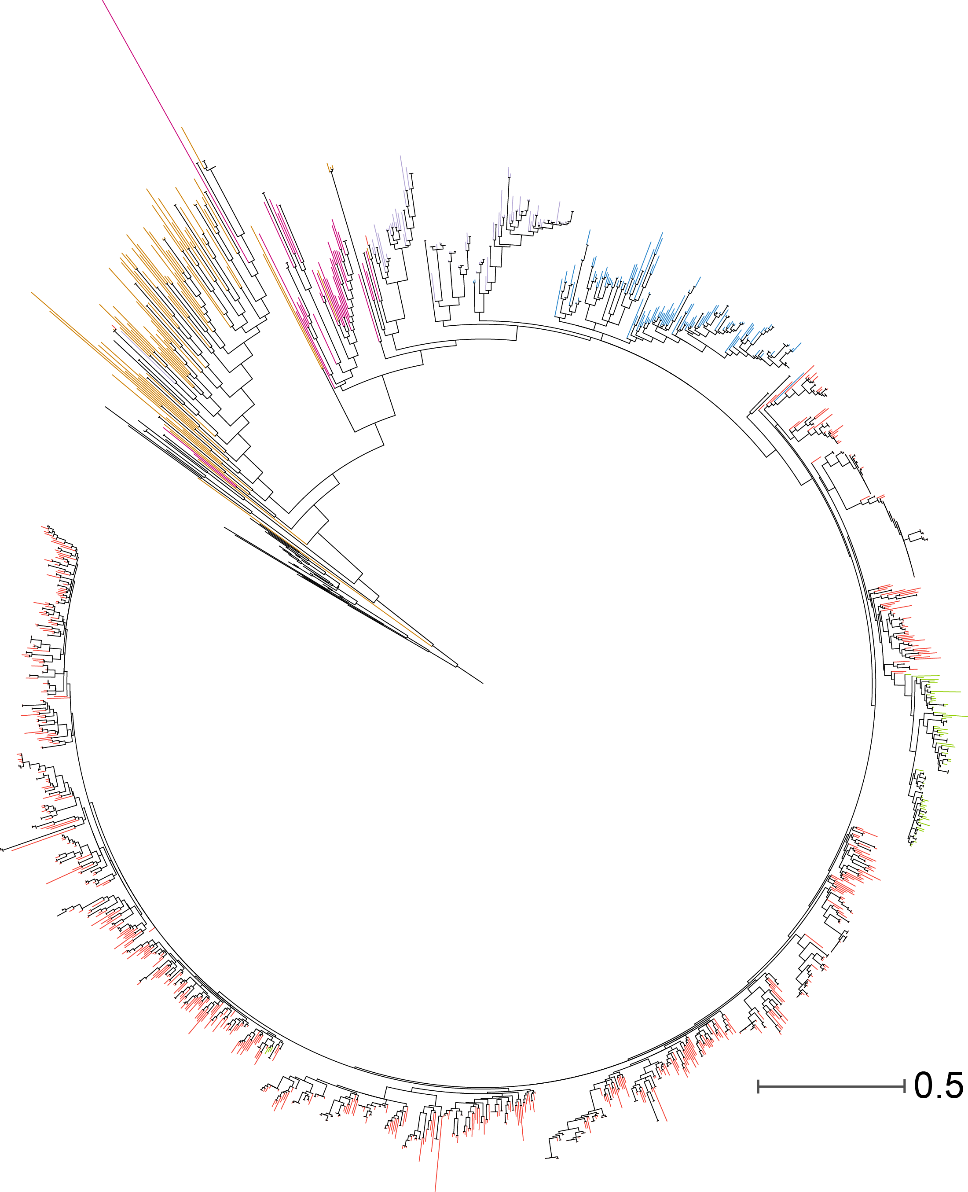


Figure S1. The phylogenetic tree of GME protein members from the 1KP database. Different colors represent different plant groups: Red: Angiosperm, Green: Gymnosperm, Blue: Ferns, Purple: Bryophyta, Pink: Charophyta, Yellow: Chlorophyta, Black: Rhodophyta.


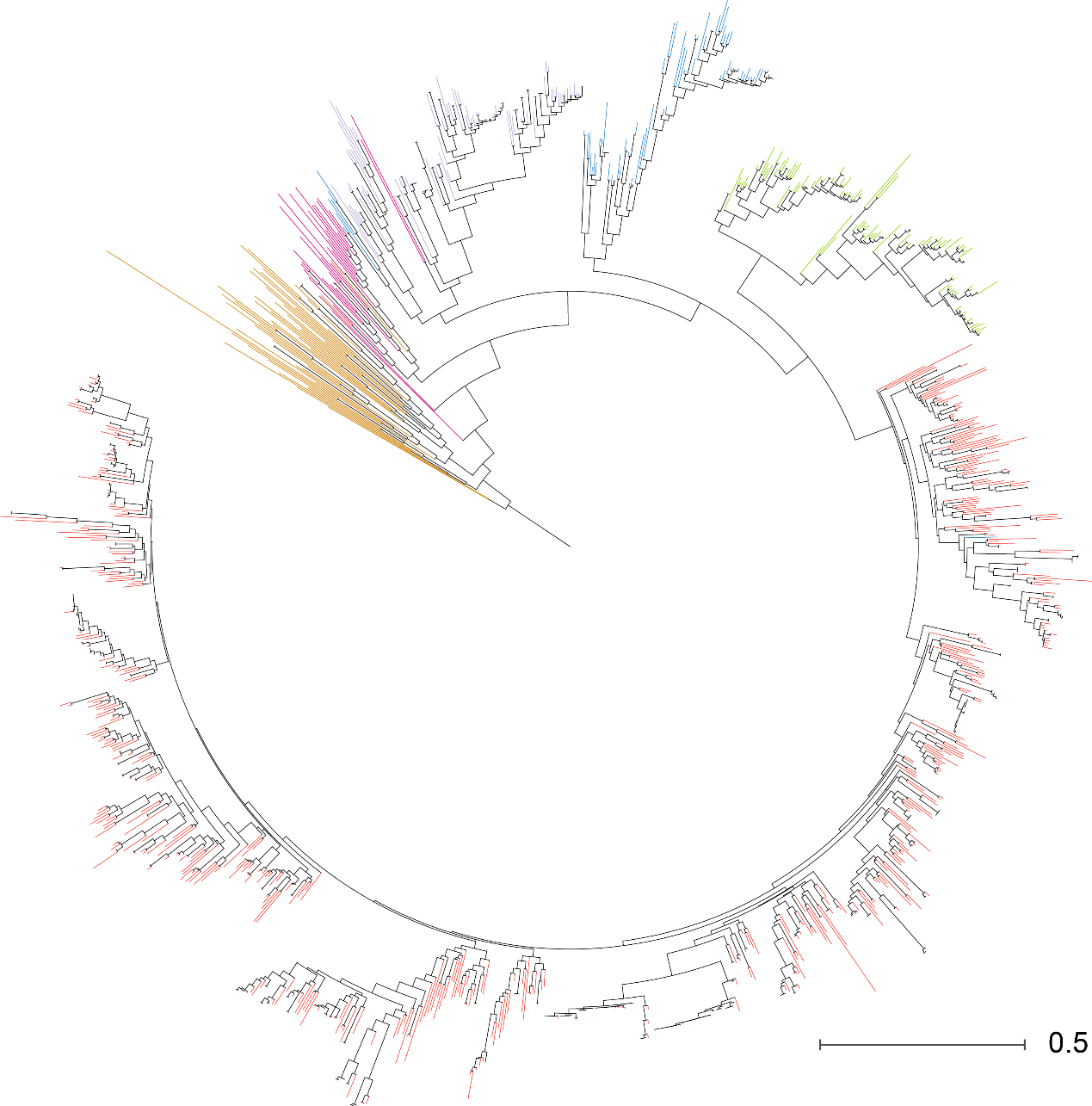


Figure S2. The phylogenetic tree of GPP protein members from the 1KP database. Different colors represent different plant groups: Red: Angiosperm, Green: Gymnosperm, Blue: Ferns, Purple: Bryophyta, Pink: Charophyta, Yellow: Chlorophyta, Black: Rhodophyta.


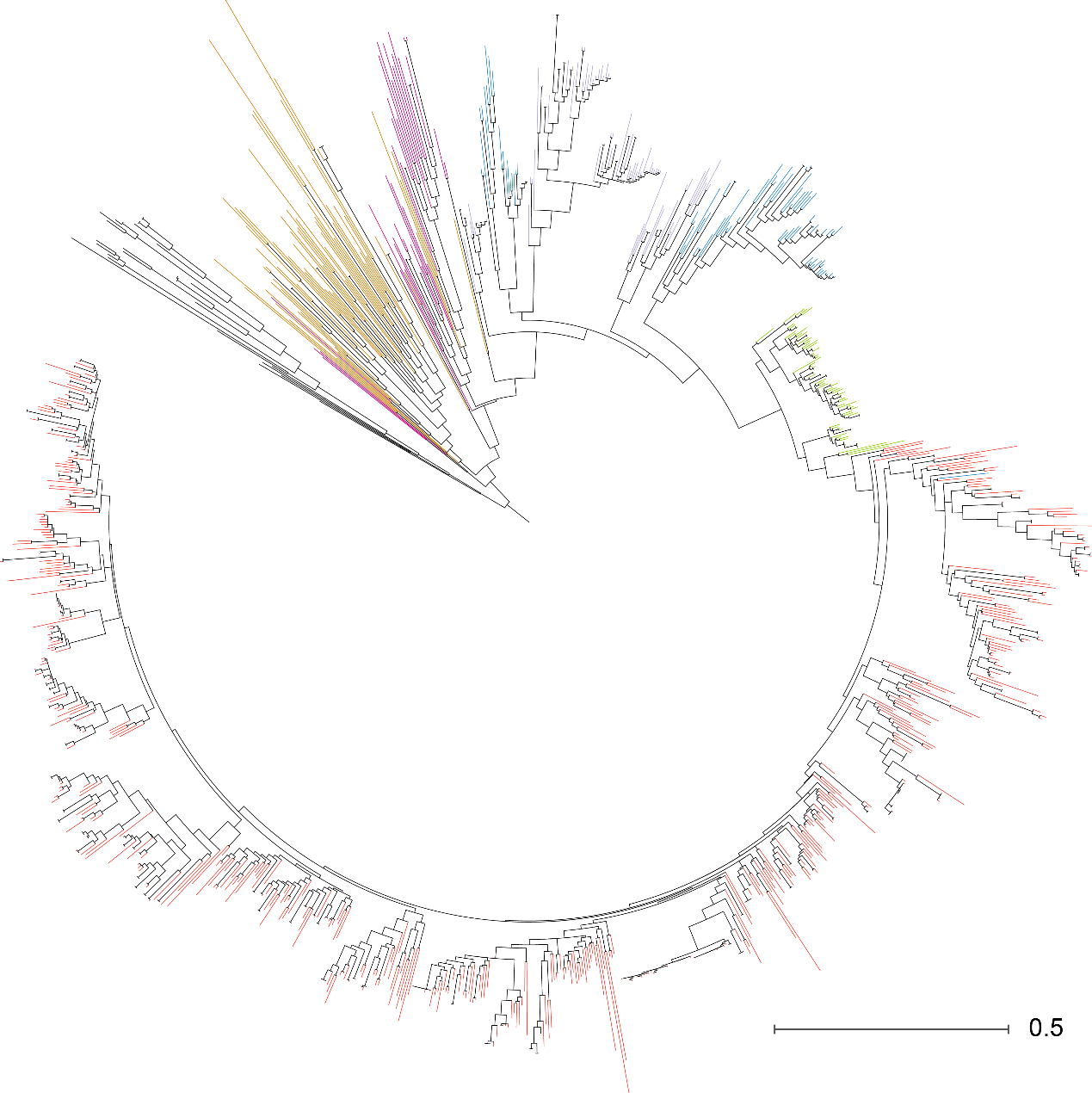


Figure S3. The phylogenetic tree of GDH protein members from the 1KP database. Different colors represent different plant groups: Red: Angiosperm, Green: Gymnosperm, Blue: Ferns, Purple: Bryophyta, Pink: Charophyta, Yellow: Chlorophyta, Black: Rhodophyta.


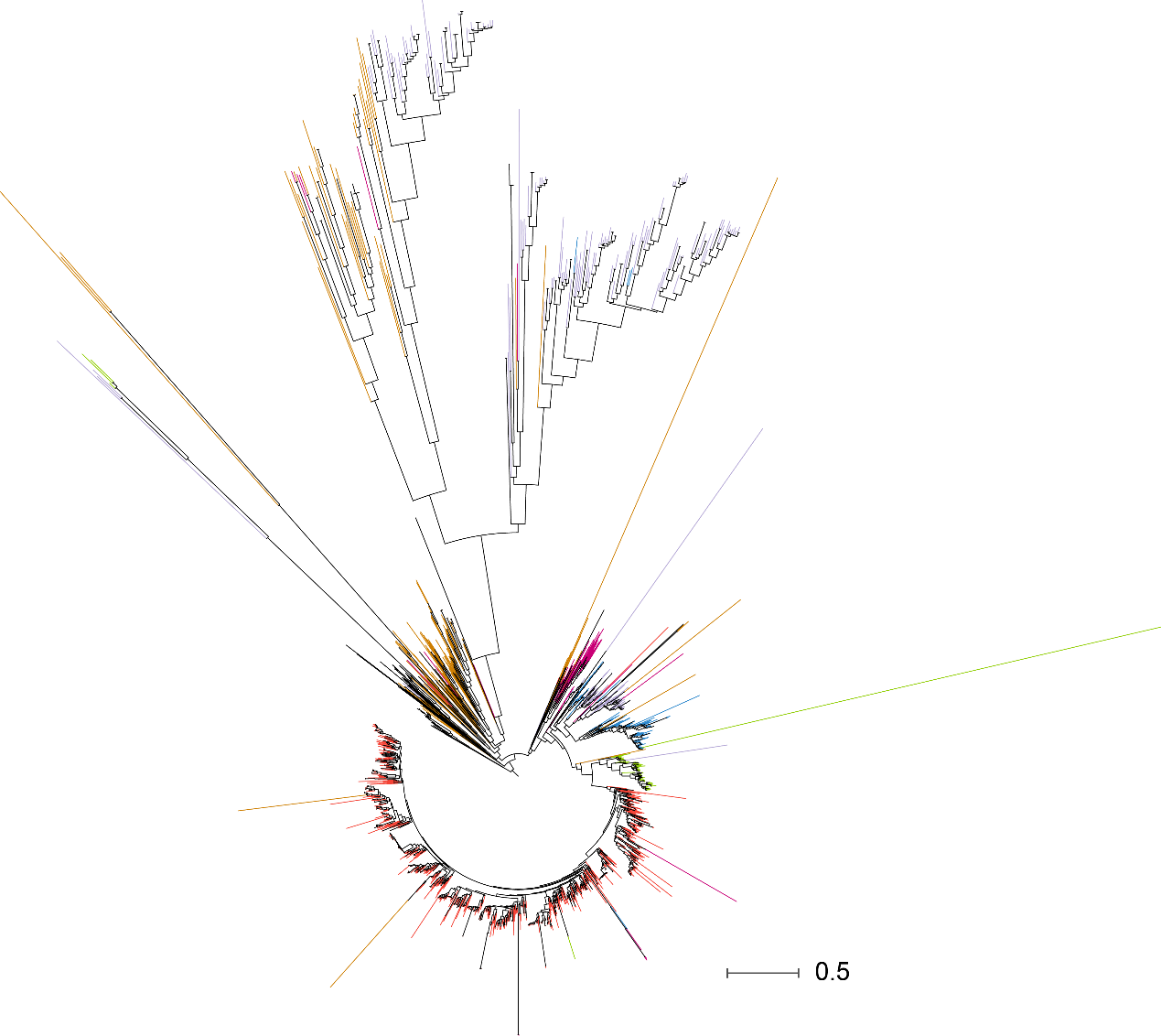


Figure S4. The phylogenetic tree of GLDH protein members from the 1KP database. Different colors represent different plant groups: Red: Angiosperm, Green: Gymnosperm, Blue: Ferns, Purple: Bryophyta, Pink: Charophyta, Yellow: Chlorophyta, Black: Rhodophyta.


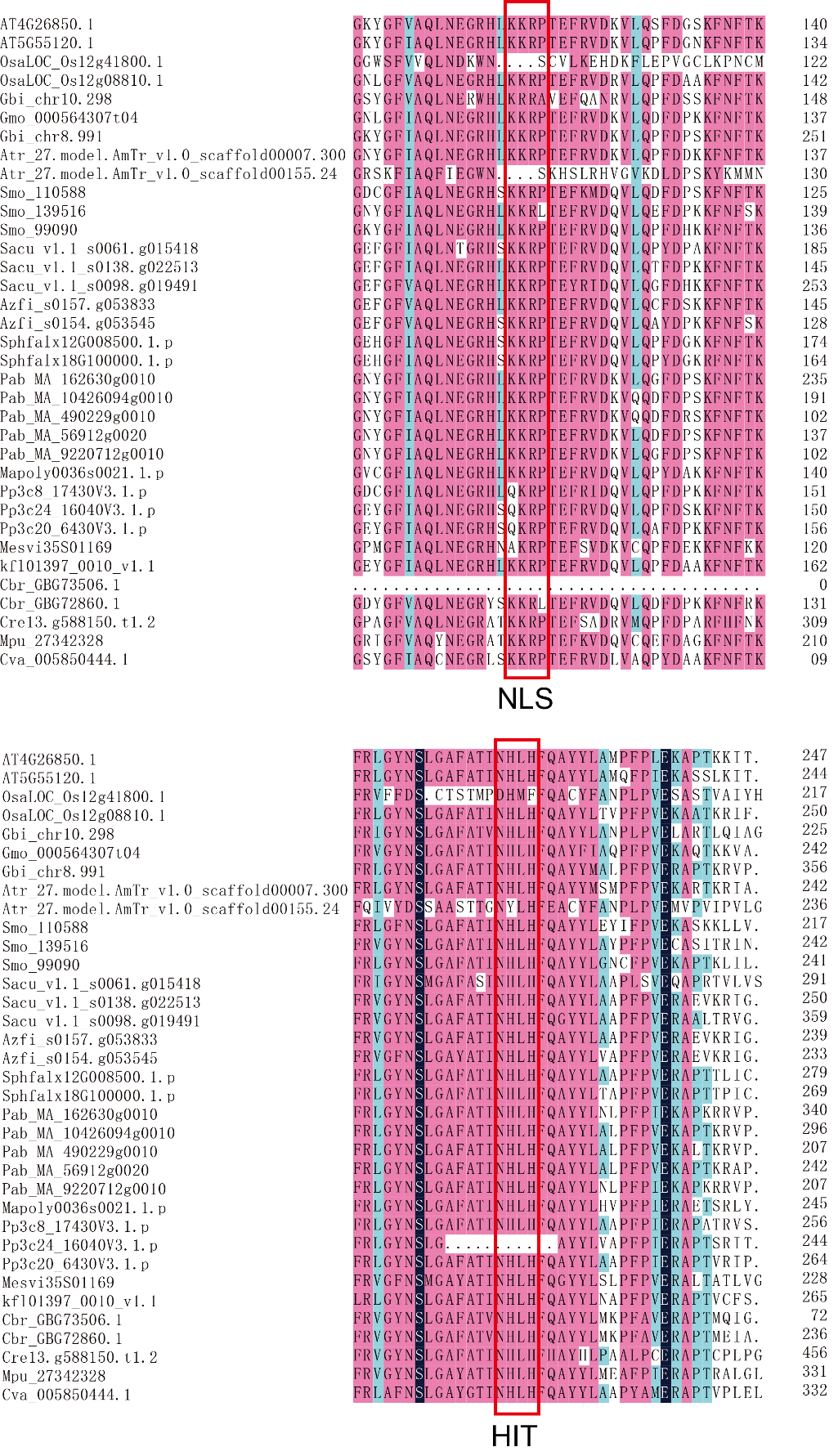


Figure S5. Multiple sequence alignment of GGP protein members from 21 core plant species. The red box indicates the NLS and HIT protein motifs of the GGP gene family.


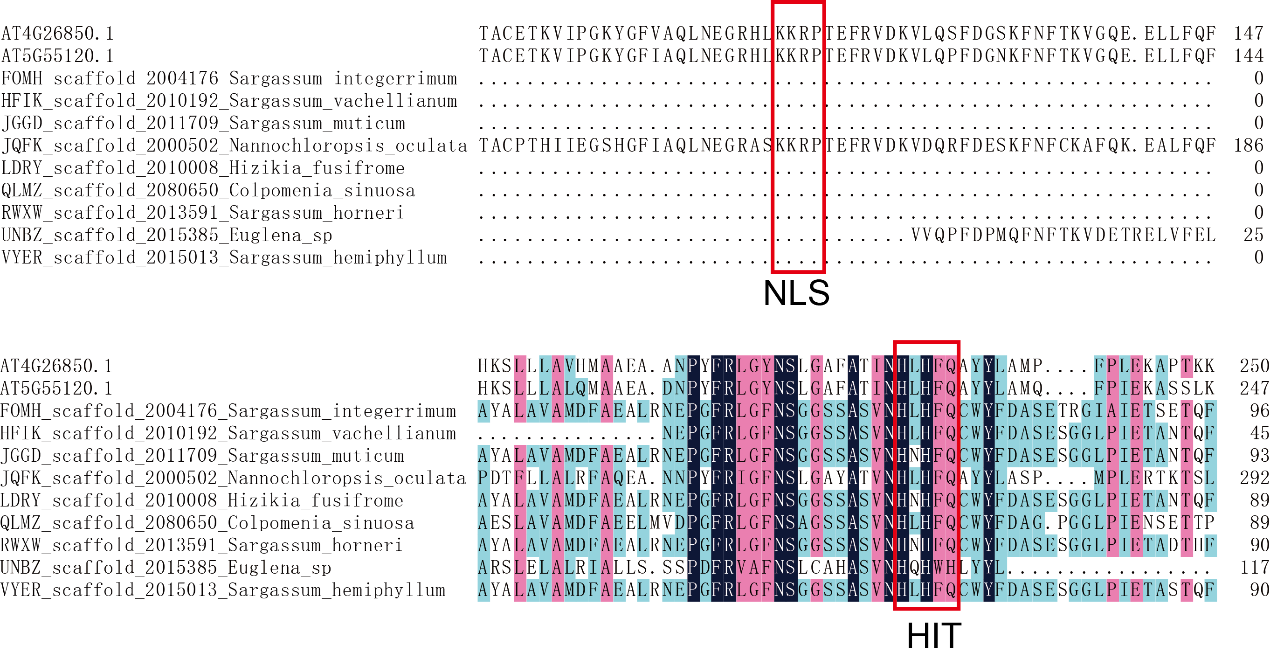


Figure S6. Multiple sequence alignment of GGP proteins between *Glaucocystis* sp. and *A. thaliana*. The red box indicates the NLS and HIT protein motifs of the GGP gene family.


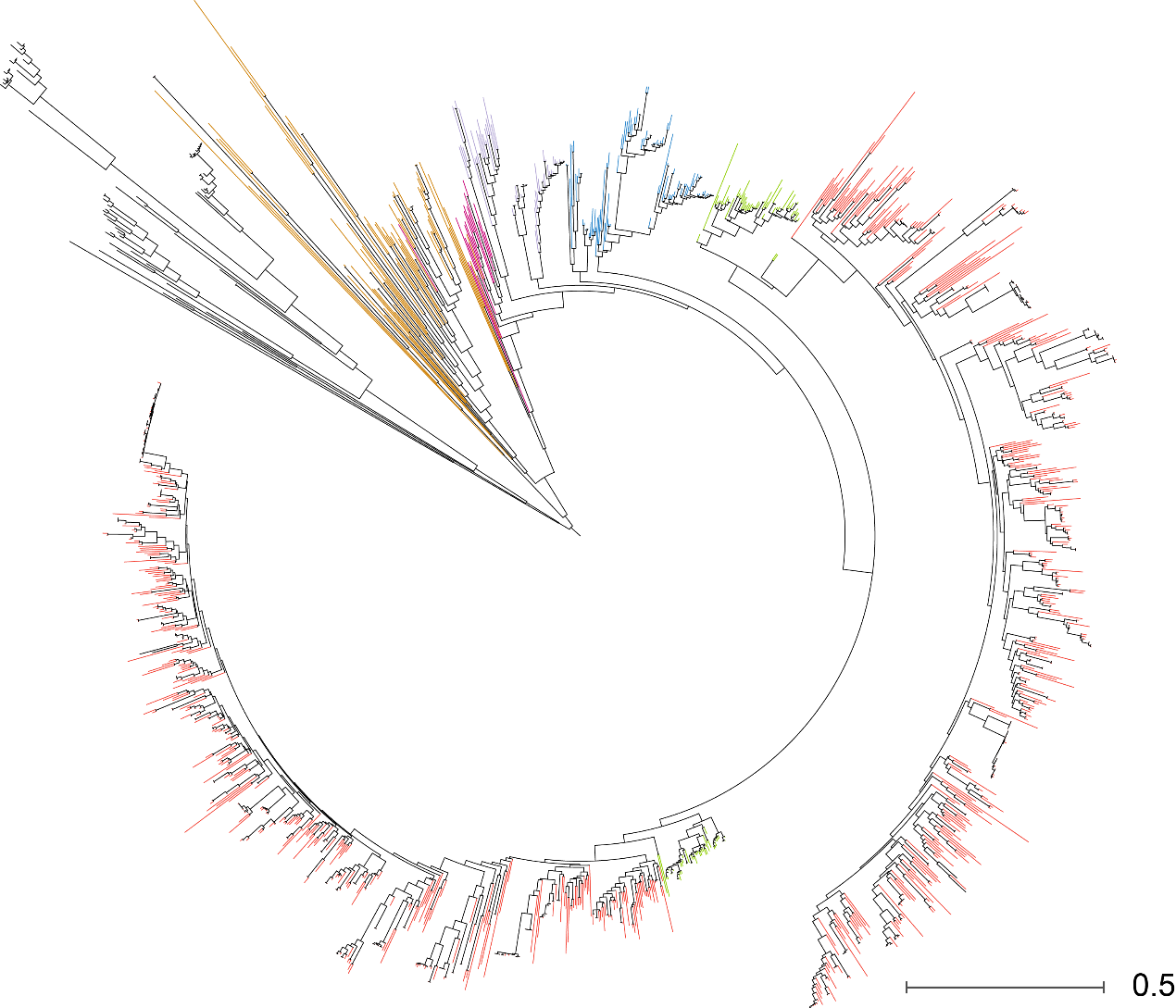


Figure S7. The phylogenetic tree of Alase protein members from the 1KP database. Different colors represent different plant groups: Red: Angiosperm, Green: Gymnosperm, Blue: Ferns, Purple: Bryophyta, Pink: Charophyta, Yellow: Chlorophyta, Black: Rhodophyta.


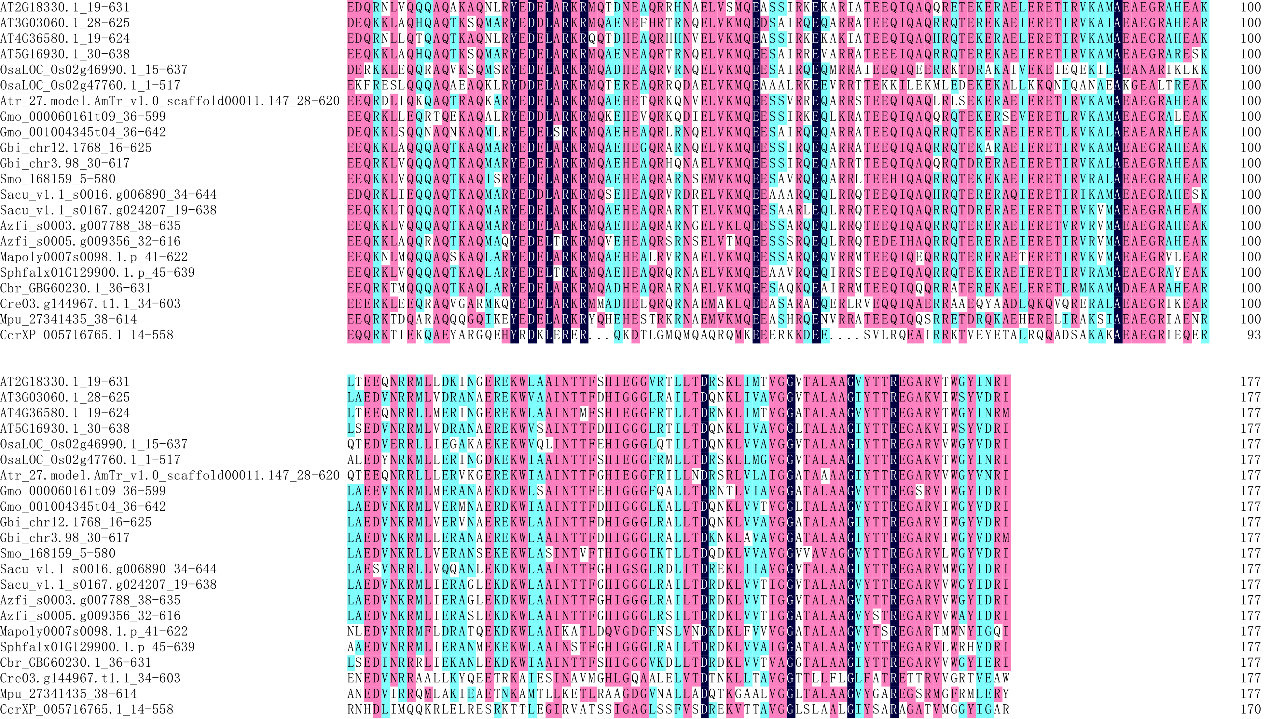


Figure S8. Multiple sequence alignment of the L-ascorbate oxidase domain in Alase protein members.


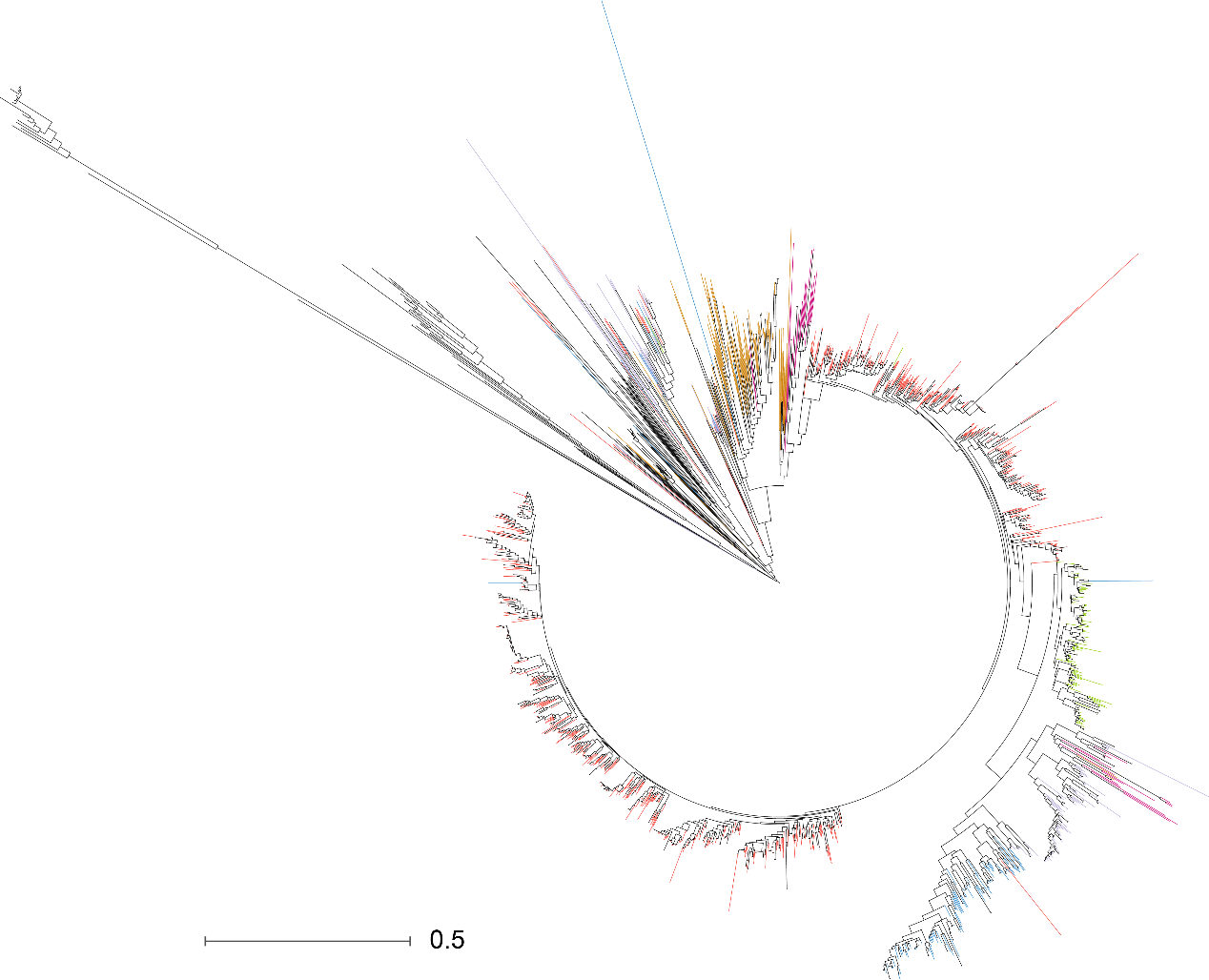


Figure S9. The phylogenetic tree of IPS protein members from the 1KP database. Different colors represent different plant groups: Red: Angiosperm, Green: Gymnosperm, Blue: Ferns, Purple: Bryophyta, Pink: Charophyta, Yellow: Chlorophyta, Black: Rhodophyta.


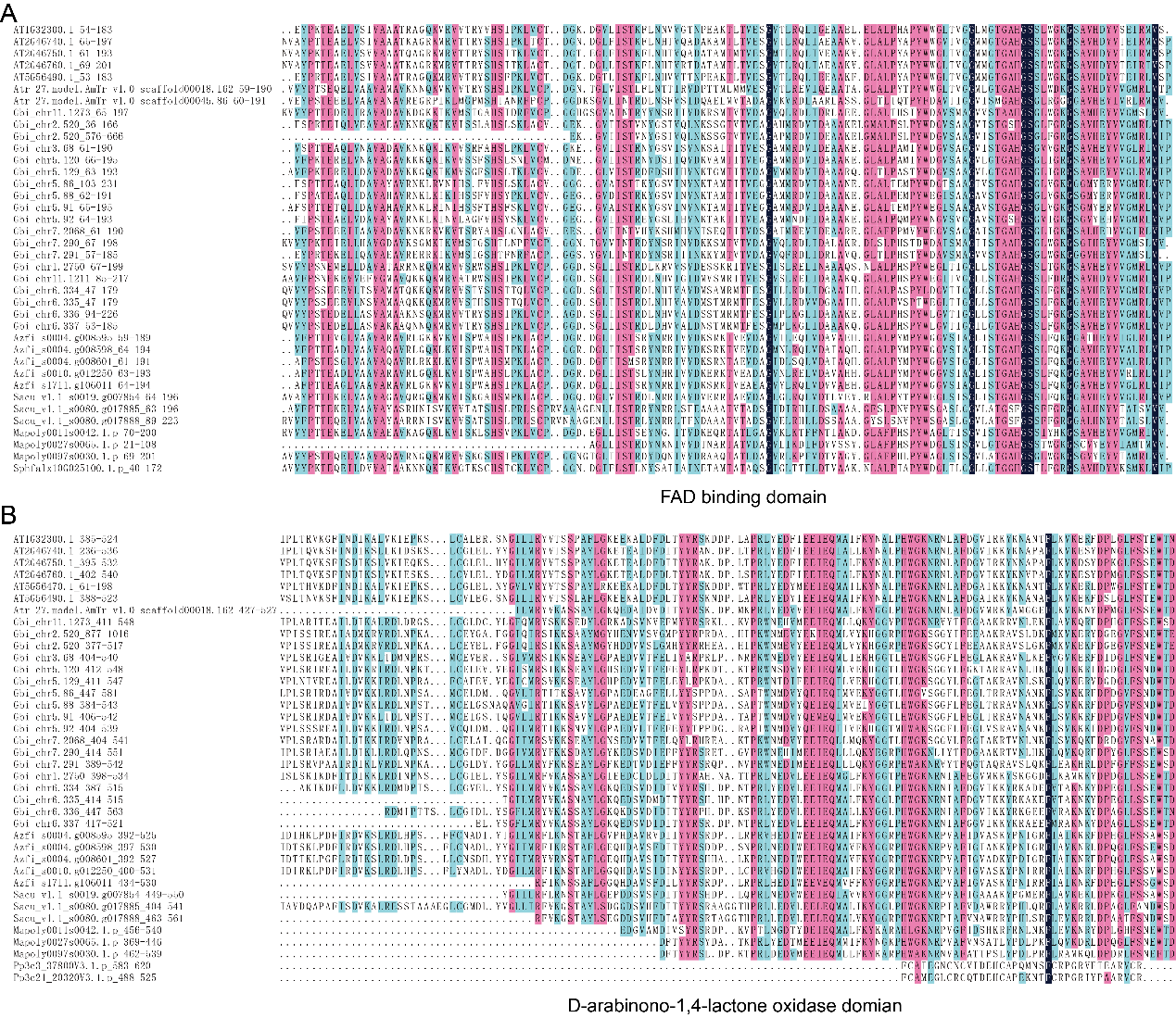


Figure S10. Multiple sequence alignment of the FAD binding domain (A) and D-arabinono-1,4-lactone oxidase domain (B) in GLOase protein members.


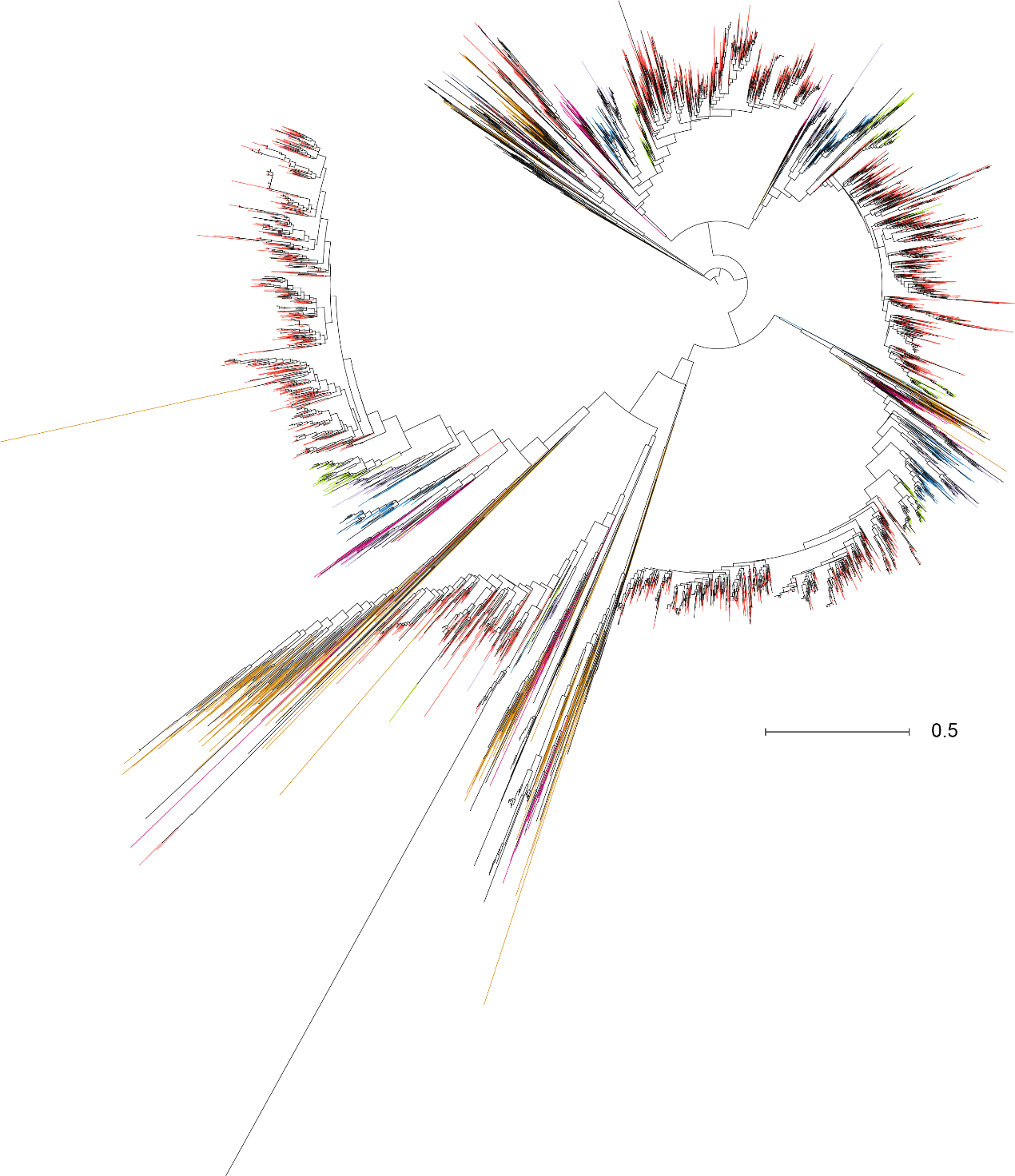


Figure S11. The phylogenetic tree of APX protein members from the 1KP database. Different colors represent different plant groups: Red: Angiosperm, Green: Gymnosperm, Blue: Ferns, Purple: Bryophyta, Pink: Charophyta, Yellow: Chlorophyta, Black: Rhodophyta.


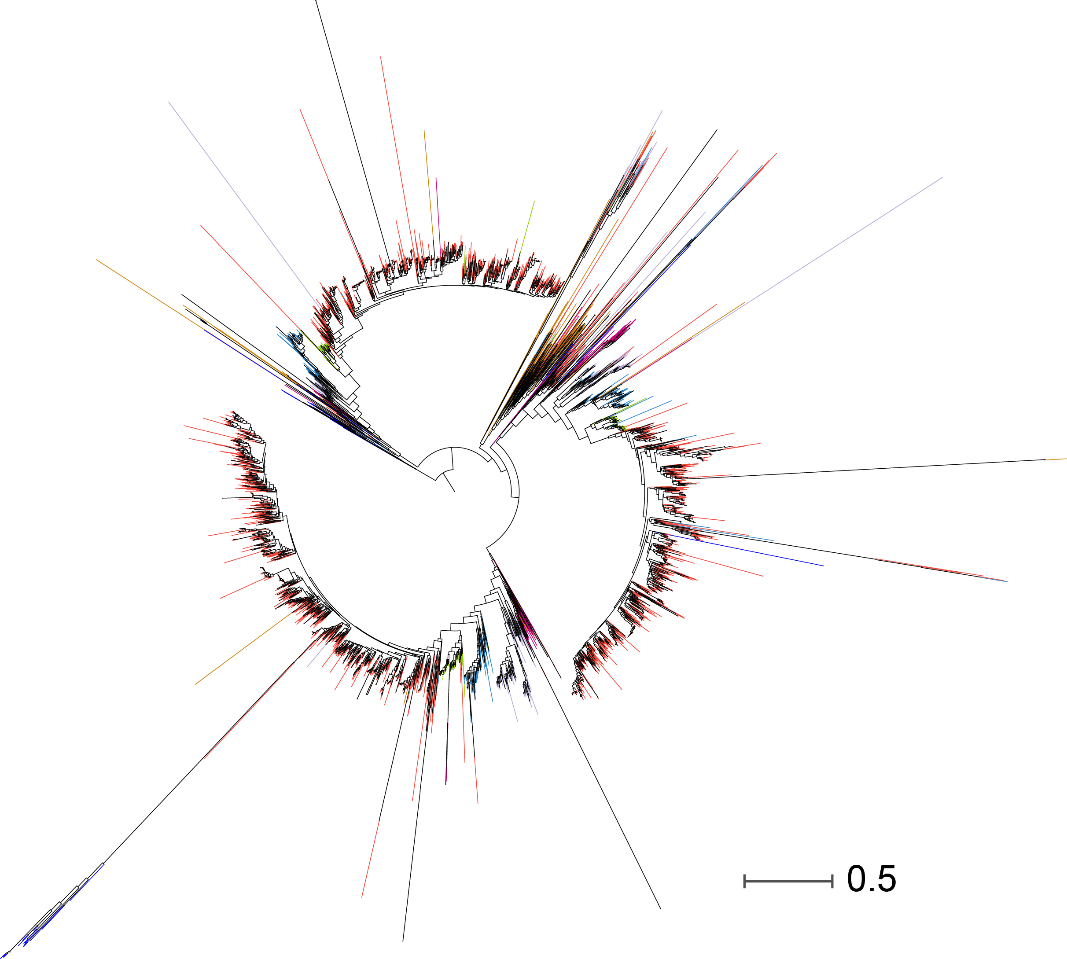


Figure S12. The phylogenetic tree of MDHAR protein members from the 1KP database. Different colors represent different plant groups: Red: Angiosperm, Green: Gymnosperm, Blue: Ferns, Purple: Bryophyta, Pink: Charophyta, Yellow: Chlorophyta, Black: Rhodophyta.


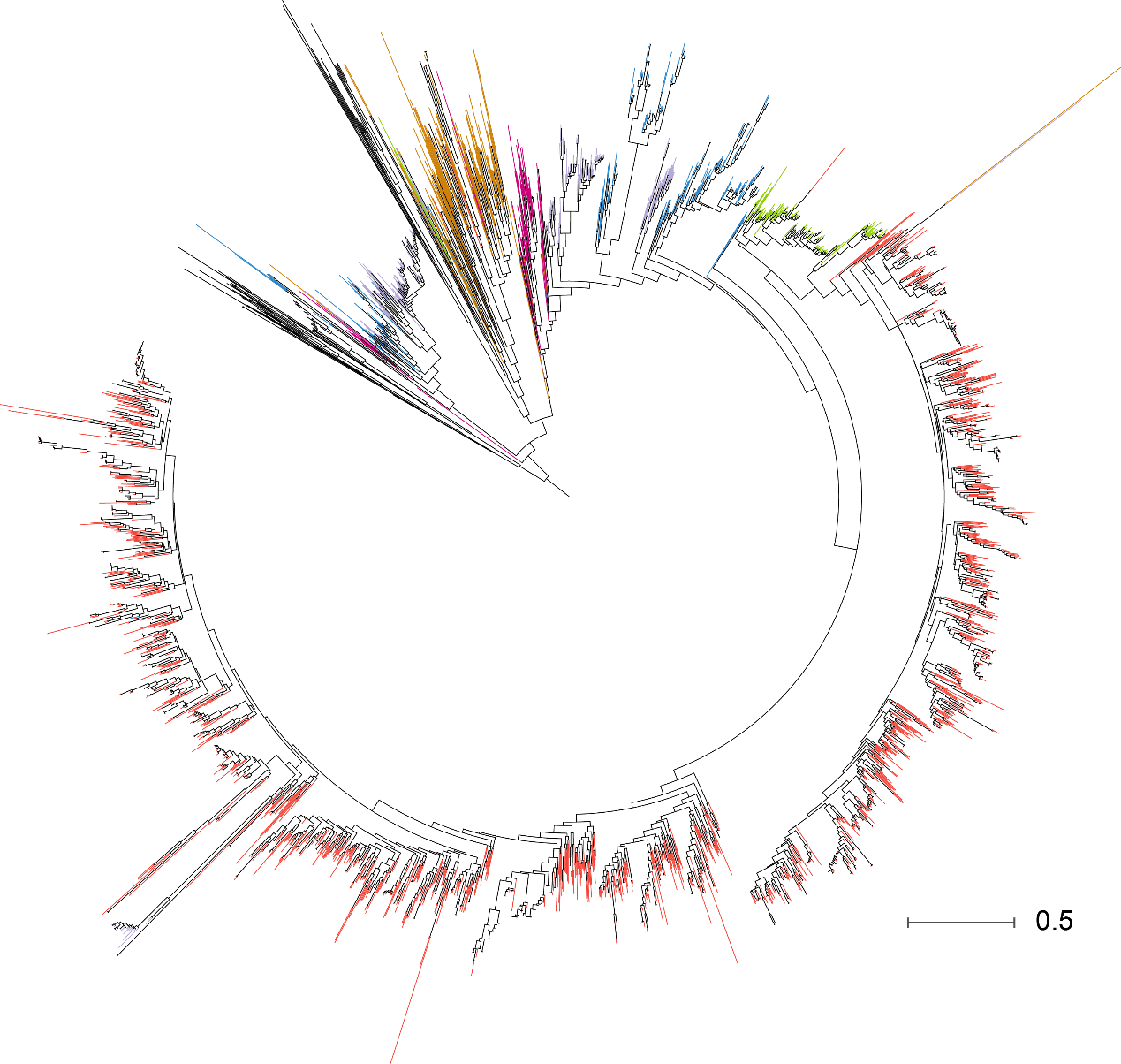


Figure S13. The phylogenetic tree of DHAR protein members from the 1KP database. Different colors represent different plant groups: Red: Angiosperm, Green: Gymnosperm, Blue: Ferns, Purple: Bryophyta, Pink: Charophyta, Yellow: Chlorophyta, Black: Rhodophyta.


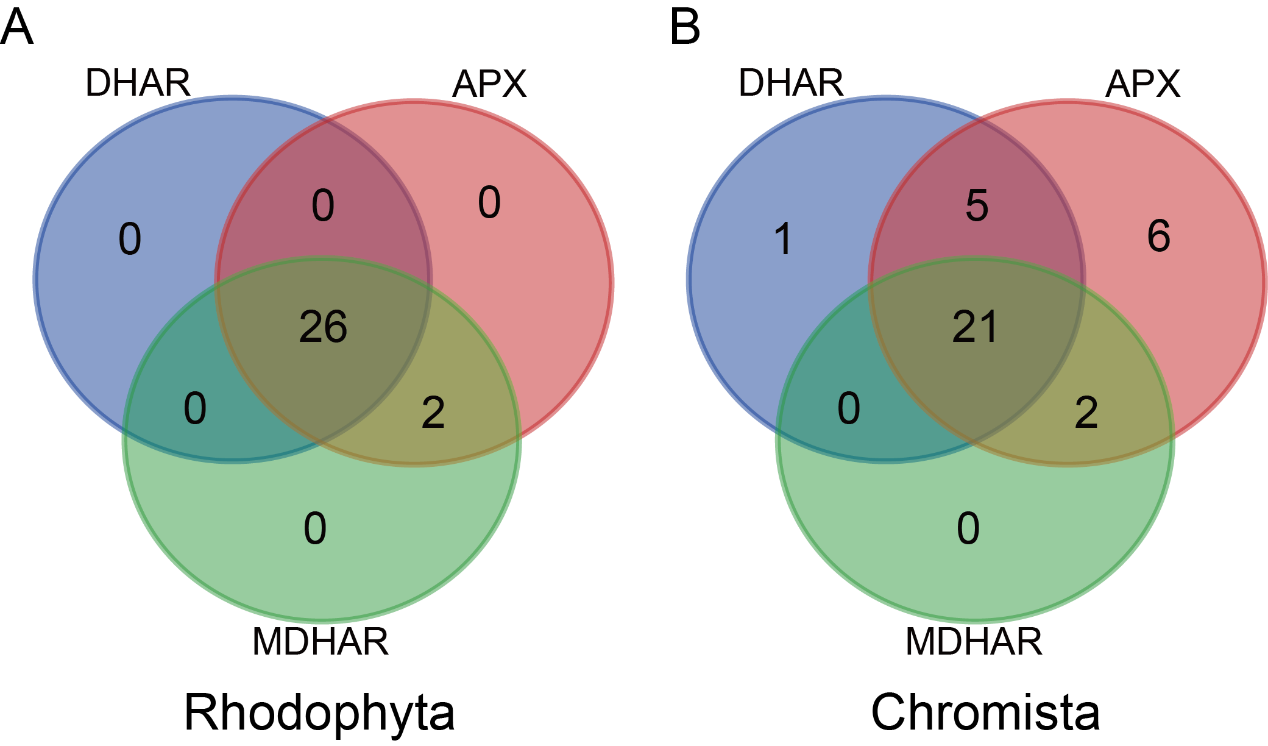


Figure S14. Clustering of core gene family members in the ascorbate cycle pathway in Rhodophyta and Chromophyte plant groups based on 1KP data.
